# Electrochemical live monitoring of tumor cell migration out of micro-tumors on an innovative multiwell high-dense microelectrode array

**DOI:** 10.1038/s41598-019-50326-6

**Published:** 2019-09-25

**Authors:** Heinz-Georg Jahnke, Agneta Mewes, Franziska D. Zitzmann, Sabine Schmidt, Ronny Azendorf, Andrea A. Robitzki

**Affiliations:** Centre for Biotechnology and Biomedicine (BBZ), Universität Leipzig, Division of Molecular Biological-Biochemical Processing Technology, Deutscher Platz 5, 04103 Leipzig, Germany

**Keywords:** Biophysical methods, Motility, Assay systems

## Abstract

Understanding of cell migration and spreading out of tumor tissue is of great interest concerning the mechanism and causes of tumor malignancy and metastases. Although there are methods available for studying cell migration on monolayer cell cultures like transwell assays, novel techniques for monitoring cell spreading out of 3D organoids or tumor tissue samples are highly required. In this context, we developed an innovative high-dense microelectrode array for impedimetric monitoring of cell migration from 3D tumor cultures. For a proof of concept, a strongly migrating breast cancer cell line (MDA-MB-231) and two malignant melanoma cell lines (T30.6.9, T12.8.10ZII) were used for generating viable micro-tumor models. The migration propensity was determined by impedimetric monitoring over 144 hours, correlated by microscopy and validated by transwell assays. The impedimetric analysis of covered electrodes and the relative impedance maximum values revealed extended information regarding the contribution of proliferative effects. More strikingly, using reference populations of mitomycin C treated spheroids where proliferation was suppressed, distinction of proliferation and migration was possible. Therefore, our high-dense microelectrode array based impedimetric migration monitoring has the capability for an automated quantitative analysis system that can be easily scaled up as well as integrated in lab on chip devices.

## Introduction

Cell migration plays an important role in many physiological processes like organ and tissue development, repair processes as well as immune system responses^1,2^. More dramatically, cell migration is the starting point for tumor metastatic spread and therefore extensively investigated for basic understanding of this pathophysiological process as well as for developing therapeutic strategies^1,2^. For 2D cultures, there are assays available to study the migratory potential of cancer cells. There are two commonly used techniques, firstly transwell assays, where cell migration through a pore-defined membrane is analyzed^3–5^ and secondly, scratch assays or wound healing assays^6–8^. One disadvantage of all these systems is the application of 2D instead of 3D cell culture models as starting point, which would provide a more complex and tumor-like test system consisting of various cell populations and oxygen and nutrition gradients mimicking the tumor environment^9,10^. The benefits of 3D cell culture systems to study migratory processes for target validation and drug evaluation have been widely published^11–13^. From a technical point of view, both assay types are based on microscopic analysis, where cells or cell covered areas have to be imaged and recognized or even prepared and stained in an end point analysis. To overcome these limitations and achieve a quantitative real-time monitoring that can be easily parallelized and automated, there were several attempts to use impedance spectroscopy for cell migration monitoring in both techniques, the scratch/wound healing assay as well as the transwell assay^14–16^. Although, these studies provided an alternative signal read-out, the used chips and systems could not overcome a common problem for all migration analysis techniques that comprises time ranges for more than some hours. All comparative analyses of cell migration between different cell populations are only consistent if cell proliferation rates of the different populations are comparable. If this is not the case, the commonly used workaround is based on the application of proliferation inhibitors like mitomycin C to suppress proliferation^7,17,18^. Although this is a widely used technique due to the lack of alternatives, treatment with proliferation inhibitors can also have impact on other cellular processes, like transcription and therefore, protein expression^19^ or may even induce cell death^7^. Therefore, artificial alterations of the cell populations can occur and cause artifacts within the comparative migration studies. There is one approach published that is based on optical image analysis and tried to overcome this problem by an initial fluorescence labeling of the cells and a differential fluorescence quantification of the cells^20^. Although this prohibits the use of proliferation inhibitors like mitomycin C, labeling of the cells with fluorescence dyes can also have unwanted side effects on the cells. Furthermore, this approach is limited to 2D cultures as starting material since the quantification of the fluorescence signal can be easily distorted by absorption in cell clusters or multilayer structures, or even differential occurance of fluorescence light absorbing molecules like pigments e.g. melanin in melanoma cells.

In this context, we wanted to use micro-tumor models in combination with innovative high-dense microelectrode arrays to first, being able to monitor the cell migration from the micro-tumor models on a 2D microelectrode array with a sufficient spatial resolution. Second, we wanted to use the cellular impedance as an additional parameter to investigate if the correlation of spatially resolved spreading and impedimetric cell signal can be used to discriminate between cellular proliferation and migration, which would be a great benefit for a comprehensive migration monitoring system.

## Results and Discussion

### Design and fabrication of the high-dense microelectrode array

**Figure 1:**
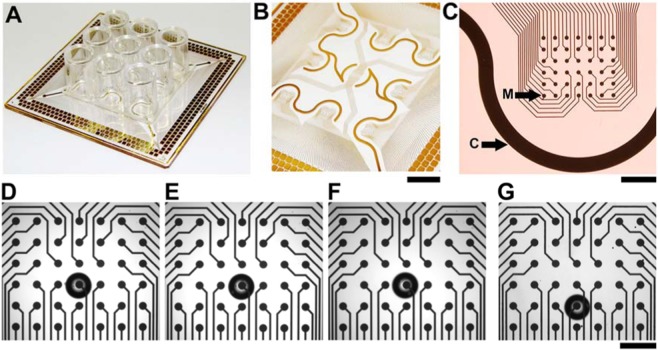
Novel multiwell high-dense microelectrode array for cell migration studies. (**A**) Complete array with 3 × 3 culture chamber in 96-well format. (**B**) Top view of the array showing electrode arrangements per well, circuit paths and contact pads (bar = 10 mm). (**C**) Magnification of one 6 × 7 microelectrode array with the microelectrodes (M) with a diameter of 100 µm and 300 µm electrode to electrode distance as well as the counter electrode (C) (bar = 1 mm). (**D**–**F**) Positioning test of a glass sphere on the array with the 96-well format culture chamber with the (**D**) initial position in the center of the microelectrode array, (**E**) after three movements of the array from and to the microscope, and (**F**) after the transport to the incubator and back to the microscope. (**G**) Using a 12-well culture chamber, the position of the glass sphere already changed after three movements of the array from and to the microscope (bar = 500 µm).

Based on the technical idea and objective to use planar electrode arrays in combination with impedance spectroscopy for an easy monitoring of cell migration from micro-tumor tissues, the demands for the electrode array were a high electrode amount and density per well for each unit and the possibility for a parallel monitoring of individual objects, preferably in an ANSI compatible multiwell format. For the proof of principle of our objective, the design is based on a 49 mm × 49 mm chip substrate that is compatible with commonly used multielectrode arrays and measurement platforms^21–24^. Taking the size of individual 3D cultures and migration capabilities into account, a multiwell system in 96-well format is suitable and was realized in a 3 × 3 well array on the chip (Fig. 1A). With respect to circuit pathways on the chip and contact pad size as well as area a maximum of 378 electrodes could be integrated, meaning 42 electrodes per well and an integrated counter electrode (Fig. 1B). With regard to sensitive cell migration monitoring within 2–6 days the electrode array was realized in a 6 × 7 array with 300 µm spacing resulting in a monitoring area of 1.5 mm × 1.8 mm (Fig. 1C). For sensitive detection of migrating single cells/cell groups, the electrode size preferably should be small. In contrast, the electrode covered area should be as big as possible to cover most of the monitoring area. As a compromise, we realized microelectrodes with a diameter of 100 µm. For defining the microelectrodes and isolation of the circuit paths we used a biocompatible SU8 photo resist that offers high cell adhesion capabilities^22,23^. To our knowledge, this innovative high-dense microelectrode array consisting of 378 microelectrodes represents the highest amount of electrodes on the used chip dimensions without using transistor based microelectrode arrays based on CMOS technique. Furthermore, 42 microelectrodes per well in a 96-well ANSI compatible multiwell format were realized for the first time in this study. Furthermore, our small scale chip with 9 wells in ANSI format offers the possibility for a future up-scaling to 96 well full format systems. Next, we focused on the positioning of the micro-tumor model cultures. With the goal to demonstrate the capability to monitor and analyze the migration out of the micro-tumor model quantitatively, we aimed to avoid additional positioning assistance by e.g. pillars^25^ or ring structures on the microelectrode array center because these would also act as migration barriers and could influence the quantitative migration analysis. From previous trials of positioning 3D cultures in the center of 24-well and 12-well plates difficulties when moving the plates are known. Therefore, we performed several trials without microelectrode array in 96-well format culture chambers (Fig. 1D–F). Interestingly, after positioning glass beads in the center of the microelectrode array under a stereomicroscope, the array could be moved from and to the microscope several times while the glass beads kept their position (Fig. 1E). Moreover, careful transfer and placement within an incubator and back to the microscope was possible without noticeable displacement of the glass beads (Fig. 1F). In contrast, when we initially attached a 12-well culture chamber on the microelectrode array it was much more difficult to keep the glass beads in the original position, when the chip was moved from the microscope and back several times (Fig. 1G). Apparently, the smaller culture volume attenuates movement introduced flows and fluid vibrations. For reproducible comparisons of different well formats and therefore, culture volumes, we placed 12-well and 96-well plates on a horizontal gyratory shaker with 60 rpm and analyzed the movement of glass beads positioned in the wells (Supplementary Figure S1, Supplementary Movie S1, S2). These experiments confirmed the attenuated fluidic flow and wave formation in the 96-well format. After these trials, we were convinced that our high-dense microelectrode array with 9 culture chambers in the 96-well ANSI format can be used for the migration experiments without additional positioning assistance structures. Nevertheless, when our array will be up-scaled to a highly parallelized 96 well full format system, it should be investigated how additionally positioning support could improve the robust handling.

### Characterization of the micro-tumor model

**Figure 2:**
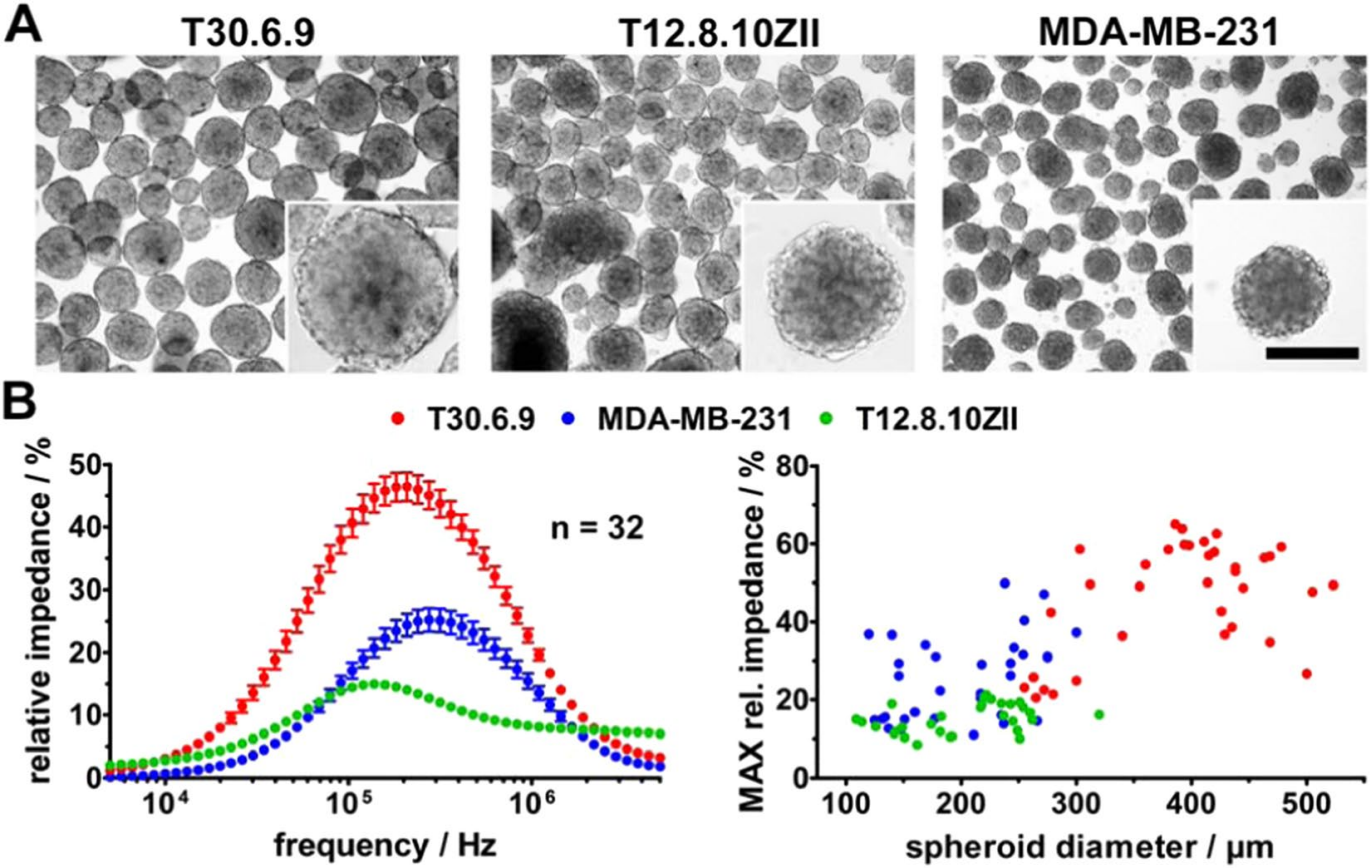
Characterization of the micro-tumor model. (**A**) Morphology of three different cell line derived spheroids after ten days of cultivation on a gyratory shaker (bar = 200 µm). (**B**) Impedimetric characterization of spheroids using microcavity arrays. The relative impedance spectra of 32 single spheroids per cell line are shown as averages. The maximum relative impedance for each spheroid was determined and correlated with the diameter of the spheroid.

For our study, we included three different tumor cell lines. As a reference for a highly migrating tumor cell line, MDA-MB-231 cells were chosen^26,27^. Additionally, we investigated two self-established melanoma cell lines (T12.8.10ZII and T30.6.9)^28^ that seem to have at least slightly different migration characteristics from microscopic observations. Initially, our micro-tumor models were characterized concerning spheroid size as well as impedimetric analysis of spheroid density with our self-developed microcavity array^28,29^. The breast cancer and malignant melanoma spheroids were cultured for ten days where their initial growth assembling and growth phase was finished. The morphology of all three cancer model cell lines was comparable and the spheroid size was in the range of 100–500 µm (Fig. 2A). The averaged relative impedance spectra of 32 individual monitored spheroids per cell line differed in their relative impedance maxima concerning amplitude and frequency (Fig. 2B). T12.8.10ZII spheroids showed the overall lowest maximal relative impedance of 14.9 ± 0.6% at approximately 137 kHz. The malignant melanoma spheroids T30.6.9 revealed the highest maximal relative impedance with 46.4 ± 2.3% at 208 kHz, whereas MDA-MB-231 spheroids showed a maximal relative impedance of 25.2 ± 1.9% at 275 kHz. Statistically significant differences concerning the relative impedance maxima appeared among others between all three cell lines (all p < 0.001). Interestingly, the spectra of malignant melanoma spheroids T12.8.10ZII that showed the lowest impedance maximum of all three cell lines, revealed increased values in the higher frequency range of 1–5 MHz. This has to be caused by an additional or extended resistor element above of the individual cell based resistors that could be the extracellular matrix resistance as it occurs in 3D cultures and tissues^28,30^.

Furthermore, the maximal relative impedance was correlated with the diameter of each individual spheroid, which revealed two different 3D tumor model populations (Fig. 2B). On the one hand, spheroids with small size and low maximal relative impedance (T12.8.10ZII) and on the other hand small (MDA-MB231) as well as larger (T30.6.9) spheroids with higher maximal relative impedance values. Important to note is that none of the individual populations followed a linear dependence between size and maximal relative impedance values. This is in line with previous characterizations of spheroid and micro-tissue cultures using the microcavity array^28,29,31^ where the maximum could be correlated with the average density of the spheroid rather than the spheroid size. The cell density as well as the size of the micro-tumor could be useful for additional parameters regarding the analysis of migration.

### Impedimetric monitoring of cell spreading originated from the micro-tumor model

**Figure 3:**
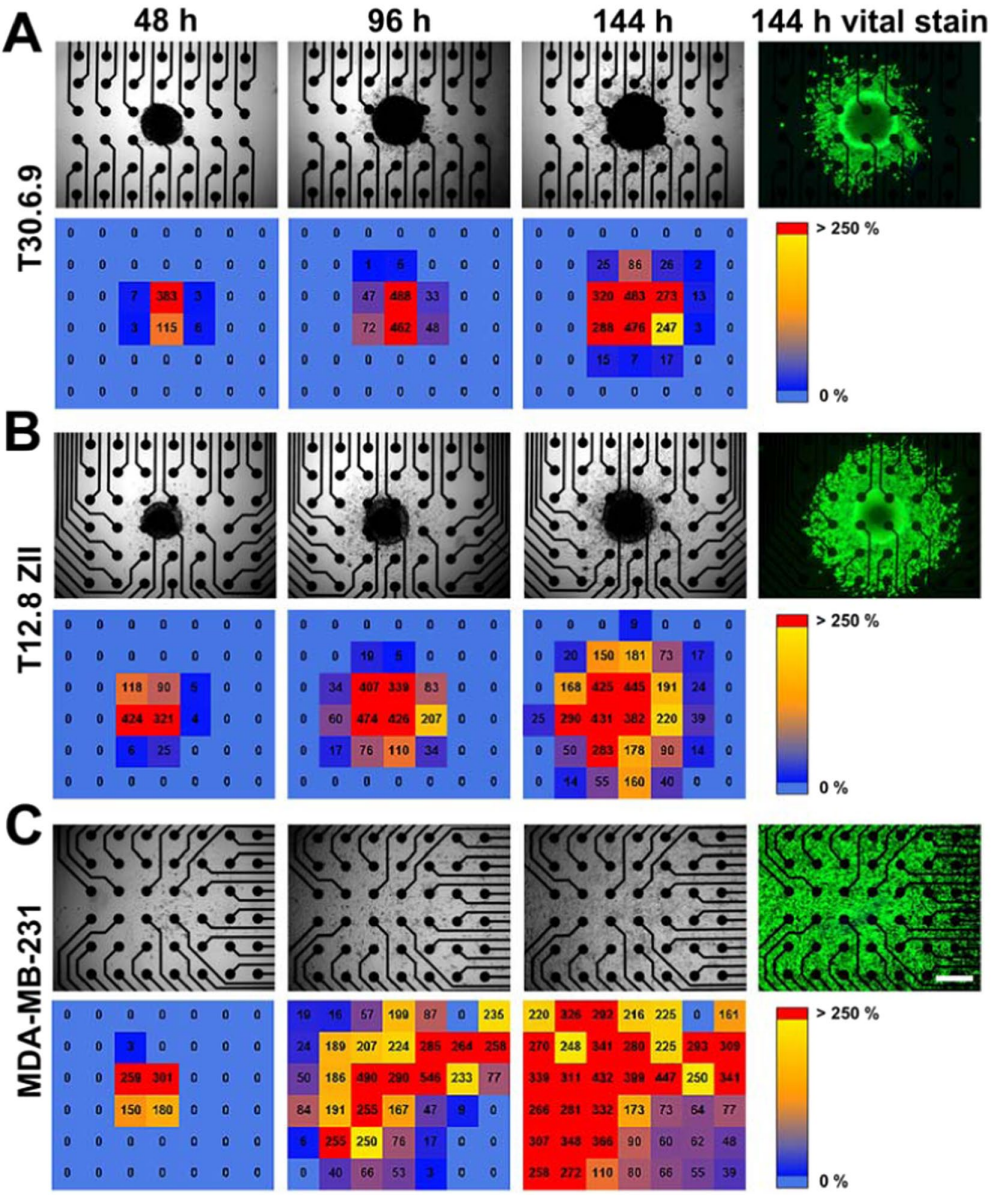
Cell migration study for the micro-tumor model on high-dense microelectrode arrays. For spheroids derived from (**A**) T30.6.9, (**B**) T12.8.10ZII, and (**C**) MDA-MB-231 microscopic images as well as mapping of the determined maximum relative impedance (color code and value) are presented. At the end of the experiment (144 hours), a vital stain (green) in combination with a dead stain (red) was performed (bar = 500 µm).

After initial characterization, the micro tumor models were investigated regarding cell migration arising from the spheroids, which was monitored with our innovative high-dense microelectrode array. Therefore, the spheroids were placed individually in the center of the microelectrode array in each well. The cell migration was first evaluated by monitoring the increase of covered electrodes that is caused by cells that migrate of the spheroid and spread over the microelectrode array (Fig. 3). For an easy visualization, we integrated a data presentation module in our analysis software that provides for each microelectrode array a color gradient scheme that represents not only if the electrode is covered or not, but moreover, is connected to the relative impedance maximum. The latter parameter gives additional quantitative information concerning the cell amount/density on the electrode. Based on this visualized time dependent spreading pattern, the highly migrating reference cell line MDA-MB-231 revealed the highest grade of spreading with almost all electrodes covered after 96 hours (Fig. 3C), followed by the T12.8.10ZII cell line with more than 60% electrodes covered after 144 hours (Fig. 3B) and finally, the T30.6.9 cell line with only 35% electrodes covered after 144 hours (Fig. 3A). For correlation, the spheroid and its migrating cells were documented microscopically and after 144 hours of monitoring a live-dead-staining was performed. All investigated spheroids showed maximal vitality levels subsequent to 144 h incubation and negligible cell death. The smallest cell spreading propensity, determined by the covered cell surface area derived from the microscopic images was observed for T30.6.9 spheroids (Fig. 3A) followed by a higher surface coverage for the T12.8.10ZII spheroids (Fig. 3B). By far the highest cell spreading were observed for the MDA-MB-231 spheroids (Fig. 3C) where the whole spheroids were already completely disassembled after 48 hours. In contrast, the T30.6.9 and T12.8.10ZII spheroids were still visible even after 144 hours. Additionally, the microscopic images revealed an increase for T30.6.9 and T12.8.10ZII spheroids that is a hint for an ongoing proliferation in the spheres. While the size increase of spheroids leads to no obvious effect on the impedimetric monitoring, the microscopic observation of cell spreading and coverage over the 2D microelectrode array perfectly match with the impedimetric analysis of the cell-covered electrodes. Thus, the impedimetric analysis is a promising alternative to the optical image based analysis like phase contrast microscopy applied here. The latter often reveal poor cell imaging contrast caused by the small-scale factor of 96 well plates and therefore, problems with e.g. light scattering and bending by the fluid meniscus occur.

### Impedimetric monitoring of cell migration correlates with transwell migration studies

**Figure 4:**
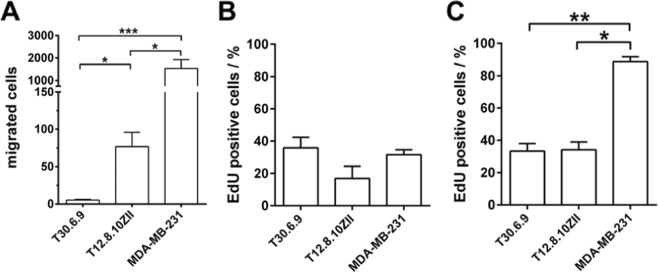
Verification of cell line dependent migration characteristics by transwell assay and analysis of proliferation in 2D and 3D cultures. (**A**) Analysis of migrating cells in the transwell assay. Cells that migrated through a porous transwell membrane insert were counted after 144 h of incubation (n = 12). (**B**-**C**) Analysis of cell line dependent proliferation by EdU incorporation assay for (**B**) 3D cultures (n = 5) and **C**) 2D cultures (n = 6). (*p < 0.05, **p < 0.01, ***p < 0.001).

For validation of the observed migration characteristics, we investigated cell migration via the commonly used transwell assay using filter inserts for 24 well plates with 5 µm pore diameter. Migrated and stained cells were counted using a microscope and data were statistically analyzed (Fig. 4A). The MDA-MB-231 cell based assays showed the highest migration rate of 1541 ± 385 cells followed by T12.8.10ZII cells (76 ± 19), whereas lowest migration was detected for T30.6.9 cells (5 ± 1). Statistically significant differences appeared among all three cell lines. Therefore, migration capabilities were comparable for both the impedimetric assay and the transwell assay. For both assays MDA-MB-231 cells displayed the highest migration rate. For the malignant melanoma cell lines the transwell assay revealed T12.8.10ZII with a higher migration rate followed by the least migrating T30.6.9 cells.

### Discrimination between cellular proliferation and migration

After we proved and validated our microelectrode array based impedimetric migration monitoring by microscopic analysis and transwell assay, we wanted to investigate if the microelectrode array based impedimetric migration monitoring can provide an improvement of the discrimination between cell proliferation and migration. If this could be achieved, there would be no need to determine proliferation rates in comparative migration studies and moreover, if there are different proliferation rates, the use of critical proliferation inhibitors like mitomycin C in each sample would no longer be necessary.

**Figure 5:**
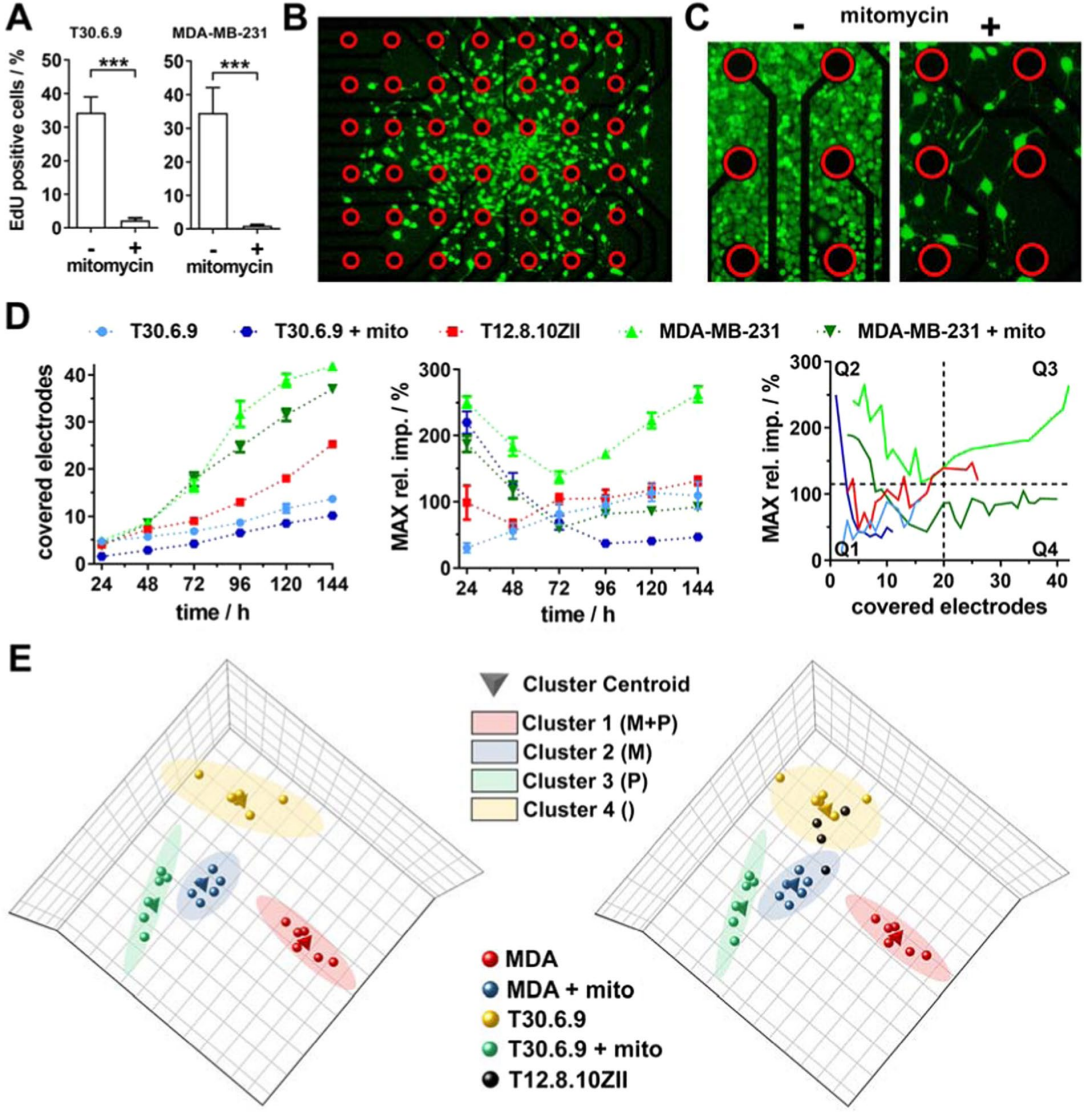
Analysis of the covered electrodes and maximum relative impedance to discriminate cellular migration and proliferation in real time. (**A**) Inhibition of proliferation by incubation with mitomycin C (n = 7). (**B**) Cell migration pattern for mitomycin C treated MDA-MB-231 on the microelectrode array (red circles mark electrodes) cells visualized by vital stain (green) in combination with a dead stain (red) after 144 hours and (**C**) a comparative magnification to mitomycin C untreated cells (red circles mark electrodes). (**D**) Covered electrodes over time reveals information of cell density on the electrodes while maximum relative impedance allows a quantification of cell density. Combining both parameters allow a classification of migration and proliferation characteristics within the four quadrants (n = 6, for T12.8.10ZII n = 4). (**E**) For an automatic classification a cluster analysis was performed for the MDA-MB-231 (MDA) and T30.6.9 cell lines with and without mitomycin treatment as reference models for migrative (M) and proliferative (P) cells. Afterwards, the T12.8.10ZII data set was included to perform the automated classification.

Therefore, we determined proliferation of all three cell lines for 3D as well as 2D cultures using the EdU cell proliferation assay (Fig. 4). While for 3D cultures (Fig. 4B) values of 35.8 ± 6.6% (T30.6.9), 16.8 ± 7.6% (T12.8.10ZII) and 31.6 ± 3.1% (MDA-MB-231) EdU positive cells could be quantified with no significant differences, for 2D cultures (Fig. 4C) values of 33.4 ± 4.6% (T30.6.9), 34.2 ± 4.8% (T12.8.10ZII) and 88.7 ± 3.1% (MDA-MB-231) EdU positive cells revealed a significantly increased proliferation for MDA-MB-231 cells in comparison to both melanoma cell lines. Since the out of the micro-tumor model migrating cells are much more comparable to a 2D culture, we decided to add mitomycin C treated MDA-MB-231 as well as T30.6.9 spheroids to our investigated panel to obtain a clear reference data set for discrimination between proliferation and migration. First, the anti-proliferative effect of mitomycin C was proved by EdU proliferation assay for T30.6.9 and MDA-MB-231 cell line (Fig. 5A). Next, microelectrode array based impedimetric migration monitoring of MDA-MB-231 spheroids was performed. While microscopic phase contrast imaging during the experiment was quite difficult, the vital stain at the experimental endpoint provided a good overview of the migrated cells (Fig. 5B). Comparing the amount of covered electrodes between mitomycin C treated and untreated spheroids still revealed a highly migratory potential for the MDA-MB-231 spheroids, but distinctly much less cells and therefore, a much lower cell density on the microelectrode array.

For an extended quantitative analysis (Fig. 5D), we used the MDA-MB-231 as well as the T30.6.9 cell line with and without mitomycin C treatment as references for low and high migration propensity. For each cell line and treatment six clusters from three independent experiments were analyzed in detail. Additionally, four clusters of the T12.8.10ZII were analyzed. First, numbers of covered electrodes were determined and traced them over time. For the first 48 hours, the number of covered electrodes was nearly the same for all cell lines and treatments (3–6) except for the mitomycin C treated T30.6.9 where the statistical analysis (Supplementary Fig. S2) revealed significant lower numbers of covered electrodes (1–3). Afterwards, there was a continuous high and significant increase for the MDA-MB-231 groups in comparison to the other two cell lines up to almost all of the 42 electrodes. The two other cell lines also showed a continuous increase, where the rate of increase of T12.8.10ZII spheres lay between the both other cell lines. The lowest increase of covered electrodes was observed for mitomycin C treated spheres up to 9–12 electrodes after 144 hours. At the end statistical analysis revealed significant differences for all groups (Supplementary Fig. S2). Furthermore, the maximum relative impedance was analyzed by calculating the median for each well and time point. The maximum relative impedance reflects the cell amount/density on the electrode and revealed a specific pattern for each cell line (Fig. 5D and Supplementary Fig. S2) with small values and a moderate increase for T30.6.9 up to 120%, while mitomycin C treatment lead to the opposite trace with high values at the beginning (220%) and a decrease to 50%. In contrast, MDA-MB-231 experienced an initial decrease from 250% to 150% after 72 hours, followed by an increase to 270% after 144 hours. Mitomycin C treatment attenuated the continuous increase after 72 hours, which reflects the suppressed increase cell numbers from the spread cells on the microelectrode array (compare with Fig. 5C)

The initial decrease for MDA-MB-231 cells with and without mitomycin C treatment within the first 72 hours could be explained by the initial fast disassembly of the spheroid on a relative small area (see covered electrode over time) followed by a fast spreading over the whole microelectrode array. When comparing the traces of mitomycin C treated MDA-MB-23, the initial course is comparable with the mitomycin C treated T30.6.9 trace followed by a more comparable course and values of the mitomycin C T30.6.9 trace. For T12.8.10ZII initial values were already in the range of 100% with a higher variance (individual values between 50–170%) but stayed relatively constant with a slight increase up to 130% after 144 hours.

With the goal to achieve discrimination between cell proliferation and migration, we plotted the maximum relative impedance (cell amount/density) against the covered electrodes (cell spreading) (Supplementary Fig. S3). The merged and averaged data sets (Fig. 5D) revealed a specific pattern for each cell line. While T30.6.9 showed low amounts of covered electrodes (<20 electrodes) correlated with low maximum relative impedance values (<100%), mitomycin C treated T30.6.9 samples revealed initial high values (>200%) with lowest amount of covered electrode (1–3) followed by a drastic decrease of maximum relative impedance (<50%) combined with the low values of covered electrodes (<12). T12.8.10ZII revealed higher amounts of covered electrodes (especially after 96 hours) correlated with maximum relative impedance values below <150%. In contrast, both MDA-MB-231 groups quickly reached high numbers of covered electrodes (>20 electrodes) correlated with high maximum relative impedance values for the untreated MDA-MB231 samples (>110%), while mitomycin C treatment lead to values below 110%. The interpretation of these patterns has taken into account that high maximum relative impedance values reflect a high amount of cells on the electrode. While only migrating cells should rapidly spread over the array resulting in a low density cell distribution, proliferating populations fill the gap between the migrating cells (see Fig. 5C). Therefore, an increased proliferation of the cell population leads to a rise of the maximum relative impedance. Based on this fact, we defined four quadrants (Fig. 5D) that reflect low migration and low cell amounts/proliferation (Q1), low migration and high cell amounts/proliferation (Q2), high migration and high cell-amounts/proliferation (Q3) and finally high migration and low cell amounts/proliferation (Q4). These characteristics are targeted especially for later time points of the migration-monitoring while for the first 24–72 hours effects like the fast adhesion and disintegration of the 3D culture can lead to values within Q2 that are not correlated with increased proliferation but cell amount. Based on the characterization (see Figs 2, 4, 5A–C), T30.6.9 and MDA-MB-231 cell lines were used as references representing low and high migration propensity and mitomycin C treatment allows the discrimination between proliferative and non-proliferative status. Thus, the four groups (T30.6.9 and MDA-MB-231 samples with and without mitomycin C treatment) were used to manually determine the threshold for the four quadrants (dashed lines in Fig. 5D). Applying this classification on the T12.8.10ZII samples, they are initially comparable to the T30.6.9 especially with regard to the maximum relative impedance values. Afterwards, they revealed a higher migration propensity than the T30.6.9 (>20 covered electrodes) but still much slower than the T12.8.10ZII samples. Maximum relative impedance values (cell density) are located at the border between Q3 and Q4, which is in correlation with the proliferation results (Figs 4C, 5A).

### Automatic classification by machine learning based clustering

A limitation of the demonstrated approach is the manual determination of the quadrant boundaries (dashed lines in Fig. 5D). Instead an automatic way to perform a classification should be realized. An advantage of the label-free and non-invasive impedimetric migration monitoring is the possibility to collect a lot of time dependent data that could be used as a large parameter set for discrimination. Actual approach for this is the application of machine learning algorithm. Therefore, we used the data set shown in Fig. 5D and performed an automatic clustering based on a principal component analysis followed by the application of a fuzzy c-means clustering algorithm. Thus, we initially used the two cell lines T30.6.9 and MDA-MB-231 as reference models for low and high migration propensity as well as with and without mitomycin C treatment to exclude or include proliferation. In accordance to the four quadrants four clusters were specified to the algorithm. As the result, each cell line/treatment group were correctly clustered (Fig. 5E). Next, to demonstrate the use of this approach to classify single new samples, data sets of T12.8.10ZII samples were included, which were mainly assigned to the cluster of cells with low migration propensity and proliferation included (T30.6.). When looking at the statistical analysis (Supplementary Fig. S2) this is verifiable since between these two groups were the lowest amount of significant differences. Although this is only an unsupervised learning algorithm on a limited dataset, this approach demonstrates how an automatic data processing could be realized based on the data sets that our novel migration monitoring provides.

Taken together, using microelectrode arrays the spatial resolution provides information of the cell spreading out of micro-tumor models and therefore, the migration propensity. Moreover, the use of microelectrode arrays provides further information of the spatially resolved cell amount. Using these two impedimetric derived parameters a discrimination between migration and proliferation can be achieved. Although the determined thresholds for the discrimination between proliferation and migration are no absolute values that could be commonly applied for other cell types or experimental settings, the use of relevant reference populations and/or comparative populations with suppressed proliferation allows to set these thresholds. Moreover, automatic classification can be achieved as demonstrated by applying an unsupervised machine learning algorithm. Especially larger data sets of reference cell lines with known migration propensities could be used as training source in supervised machine learning models. In combination with e.g. further increase of microelectrode density a reliable and more detailed classification could be realized. Based on this, extended migration studies and screenings could be performed allowing discrimination between cellular proliferation and migration without the use of critical proliferation inhibitors for each sample and extended correlative molecular biological analysis. Hence, our high-dense microelectrode array based impedimetric migration monitoring provides a clear progress in comparison to previous approaches where only averaged values of cell coverage were analyzed^15^.

Additionally, the capability for quantitative analysis of cell migration of 3D cultures offers a great opportunity for a technical extension regarding an implementation in a microfluidic lab on chip simulating the tumor environment monitored in real time. Furthermore, the presented impedimetric monitoring of cell spreading on a 2D surface by a planar microelectrode array could perhaps be extended to monitoring in 3D matrices of small height^32^.

Taken together, the presented innovative high-dense microelectrode arrays allow the parallel migration monitoring on individual 3D cultures by impedance spectroscopy. For proof of principle, we used different cancer cell line derived spheroids as micro-tumor models. The use of 3D instead of 2D cell cultures and therefore, the development of appropriate monitoring techniques and assays for analyzing tumor cell spreading and migration out of it more closely recapitulate the *in vivo* situation. Thus, our high-dense microelectrode array based impedimetric migration monitoring offers the possibility for direct analysis of e.g. tumor biopsy material from patients with regard to their migratory potential. Moreover, the microelectrode array based impedimetric monitoring reveals extended information on the temporally and spatially resolved cell spreading as well as cell amount and therefore, allows a discrimination of cellular proliferation and migration. Although, this study provides a first proof of principle, the demonstrated technique can be easily extended and integrated in e.g. 96-well full format plates for screening purposes or even into lab on chip devices. Thus, combination with microfluidic systems for the automated positioning and environment control as well as integrated migration and proliferation analysis for extended lab on chip applications are possible.

## Methods

### Cell culture

Breast cancer cells MDA-MB-231 (ATCC, USA) and human malignant melanoma cell lines T30.6.9 and T12.8.10ZII^28,33^ were cultivated in RPMI 1640 supplemented with 10% fetal bovine serum, antibiotics (2 U/mL penicillin, 0.2 µg/mL streptomycin and 20 µg/mL gentamycin) and 2 mM GlutaMAX (all from Life Technologies, Germany). Medium was changed every 2–3 days. For 3D cell cultures 0.5 million (T30.6.9) or 1 million cells (MDA-MB-231, T12.8.10ZII) were seeded in 2 ml medium in non-adherent six well plates (Greiner Bio-one, Germany) and cultivated on an orbital shaker (72 rpm) for 10–13 days. All cell cultures were maintained at 37 °C and 5% CO_2_. For suppressing proliferation in MDA-MB-231 spheroids, 10 µg/ml mitomcyin C (Sigma-Aldrich, Germany) was applied for 3 hours the day before migration experiments started.

### Microcavity and high-dense microelectrode array fabrication

Microcavity arrays for the analysis of spheroid cultures were produced as previously described^34^. High-dense microelectrode arrays in 96 well-scaled plates were produced in clean room class 100 environment by lift-off technique. Briefly, borofloat glass substrate surfaces (49 × 49 × 1 mm, Goettgens Industriearmaturen; Germany) were cleaned by piranha etching followed by intensive washing with ultrapure water. For structuring, substrates were spin-coated with negative resist (AR-N 4340, Allresist, Germany) creating a coating thickness of 2 µm. After soft baking, photo mask structure (Cr mask, Compographics, Germany) were used to transfer the chip layout to the substrate by using a MA6 mask aligner (350–405 nm, exposure time 8 s; Süss MicroTek, Germany). After a post exposure bake step (60 s, 95 °C) structures were developed in AR-N 300–475 (Allresist, Germany), rinsing and drying substrates at 95 °C. After deposition of an indium tin oxide adhesion layer (50 nm; EvoChem, Germany) the gold layer was deposited (500 nm, Junker Edelmetalle, Germany) using a sputter coater (CREAMET 500, Germany). Negative resist and on top metal were removed by incubation in acetone followed by extensive washing with isopropanol and ultrapure water prior to dehydration at 200 °C for at least 30 min. For defining the microelectrode structures, a SU-8.2 (Micro Resist Technology, Germany) insulation layer of 2 µm thickness was added via spin coating, pre-bake (1 min 65 °C), soft-bake (1 min 95 °C), 4 s UV exposure, post exposure bake (1 min at 65 °C followed by 1 min at 95 °C) and 1 min development in mr-Dev 600 (MicroChem, Germany). Finally, the high-dense microelectrode arrays were cleaned in ultrapure water, spin-dried and dehydrated at 95 °C. For cleaning and making the surface more hydrophil a plasma cleaning step at 500 mA for 450 s in argon atmosphere (CREAMET 500, Germany) was added. Finally, a 3 × 3 culture chamber in 96-well format (Greiner Bio-One, Germany) was bonded with epoxy resin (Epoxy Technology, Germany) onto the microelectrode array.

### Impedance spectroscopy

Spheroid cultures were measured using self-developed microcavity arrays with pyramidal cavities (edge length 200 µm, depth 100 µm) in combination with a self-developed multiplexer system^24^ and the high precision impedance analyzer ISX-3 (Sciospec Scientific Instruments, Germany). Impedance spectra were recorded from 5 kHz to 5 MHz (51 points, 100 mV amplitude). For cell migration on high-dense microelectrode arrays, the array surface was coated with collagen I (Life Technologies, Germany) for 1 hour. Afterwards, individual spheroids were placed centrally in each well. Impedance spectra were recorded for 144 hours from 5 kHz to 5 MHz (41 points, 10 mV amplitude) using an extended multiplexer system with 378 channels and the high precision impedance analyzer ISX-3. Raw data were analyzed and processed with the self-developed software IDAT v3.6. The relative impedance (extracted cell signal) was calculated as follows: (IZI with cells - IZI without cells) / ZI without cells × 100%. Finally, vital staining was performed by 10 min incubation with 2.5 µM calcein AM (eBioscience, Germany), 1 µM propidium iodide (VWR, Germany) and 1 µg/mL Hoechst 33342 (Life Technologies, Germany).

### Transwell-migration assay

For 2D cell migration analysis, 24 well plate hanging cell culture inserts with 5 µm pore diameter (Merck Millipore, Germany) were applied with 150,000 cells on top of the membrane in 300 µl medium and additional 500 µl medium in the plate bottom. Cell culture was maintained for 144 h with subsequent cell staining (10 min 2.5 µM calcein AM and 1 µg/ml Hoechst 33342) and microscopically based counting of migrated cells on a fluorescence microscope (TE2000, Nikon, Germany).

### Cell proliferation analysis by flow cytometry

To determine cellular proliferation, cells were cultured as monolayers in collagen I coated 6-well plates up to a confluence of about 70% and as 3D cultures in non-adherent 6-well plates for 10–13 days that represents the initial status of the migration experiments. All cell cultures were incubated with 10 µM EdU (Life Technologies, Germany) for 16 hours prior to harvesting. Afterwards, cultures were dissociated by incubation with trypsin-EDTA (0.25%, Life Technologies) for 5–10 minutes followed by a mechanical dissociation. Then single cells were fixed for 20 min with 4% formaldehyde (Merck, Germany) in PBS. Samples were stored in 0.1% Triton PBS and stained for flow cytometry using the Click-iT EdU Alexa Fluor 488 assay kit (Life Technologies, Germany) and counter stained for 10 min with 7-AAD (BD Biosciences, Germany). Stained cells were analyzed using a BD FACS Calibur (BD Biosciences, Germany).

### Statistical analysis and automatic clustering

For statistical analyses in Prism 5.02 (Graphpad, USA), firstly the data were checked for normality distribution (D’Agostino and Pearson omnibus test) and secondly (no normality distribution) the data were analyzed with Kruskal Wallis and Dunns post hoc test. Presented data are mean values ± standard error of the means (SEM). For automatic clustering a self-developed program based on the LabVIEW machine learning toolkit (National Instruments, Germany) was used. For clustering, time dependent (six time points) values of covered electrodes and maximum relative impedance were used as 12 input parameters for a principal component based reduction to three dimensions. Afterwards, an unsupervised machine learning clustering algorithm (fuzzy c-means) was used for automatic classification.

## Supplementary information

Supplementary Information

Supplemetary Movie S1

Supplementary Movie S2
