# Supplementary material for: Electrochemical live monitoring of tumor cell migration out of micro-tumors on an innovative multiwell high-dense microelectrode array
Source: Sci Rep. 2019 Sep 25;9:13875. doi: 10.1038/s41598-019-50326-6 (PMC6761180; doi:10.1038/s41598-019-50326-6)
Supplement: Supplementary file 1 — Supplementary Information [file 41598_2019_50326_MOESM1_ESM.pdf]

SUPPLEMENTARY INFORMATION

Electrochemical live monitoring of tumor cell migration out of micro-tumors on an innovative multiwell high-dense microelectrode array

Heinz-Georg Jahnke, Agneta Mewes, Franziska D. Zitzmann, Sabine Schmidt, Ronny Azendorf and Andrea A. Robitzki

Supplementary Figures

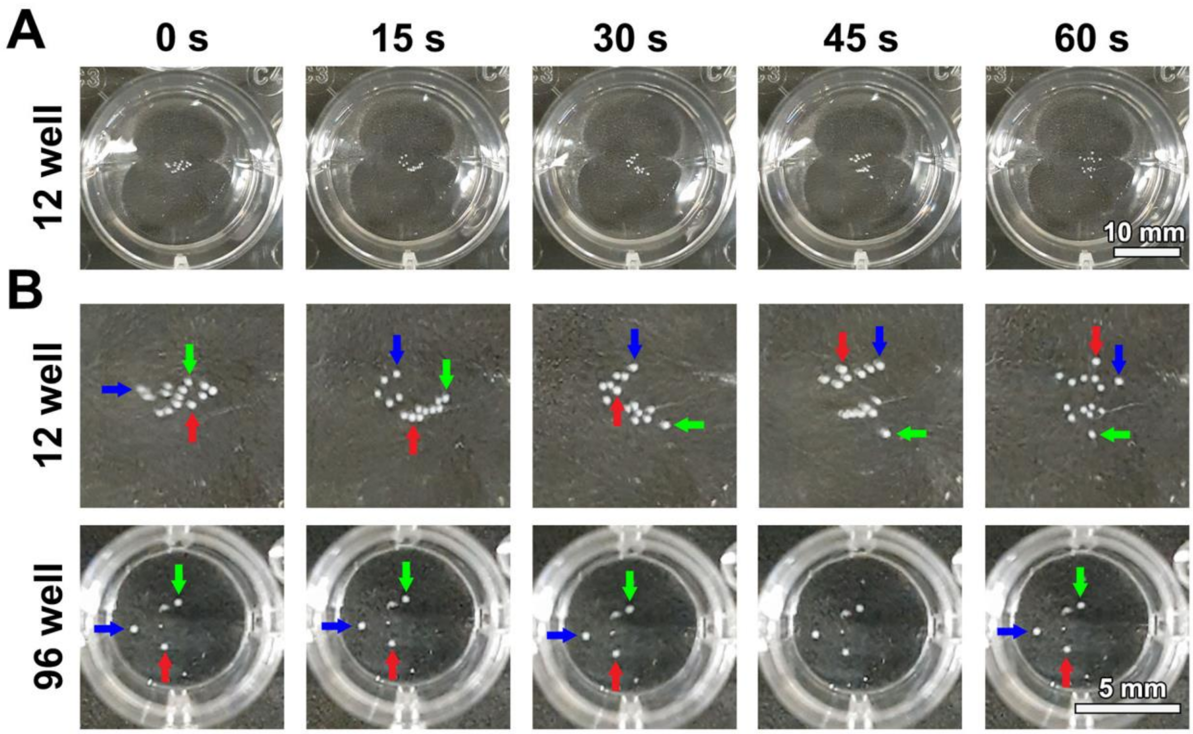

Supplementary Figure S1: Comparison of particle movement in 12-well and 96-well format. For a reproducible comparison 12-well and 96-well plates were placed on a gyratory shaker with 60 rpm with the standard culture liquid volume of 1 ml for the 12 well plate and 200 µl for the 96 well plate. Glass spheres were placed within the well and monitored for one minute. **(A)** Overview for a single well of the 12 well plate and (B) a comparable magnification for both plate sizes. Individual glass spheres are marked with coloured arrows and tracked over time.

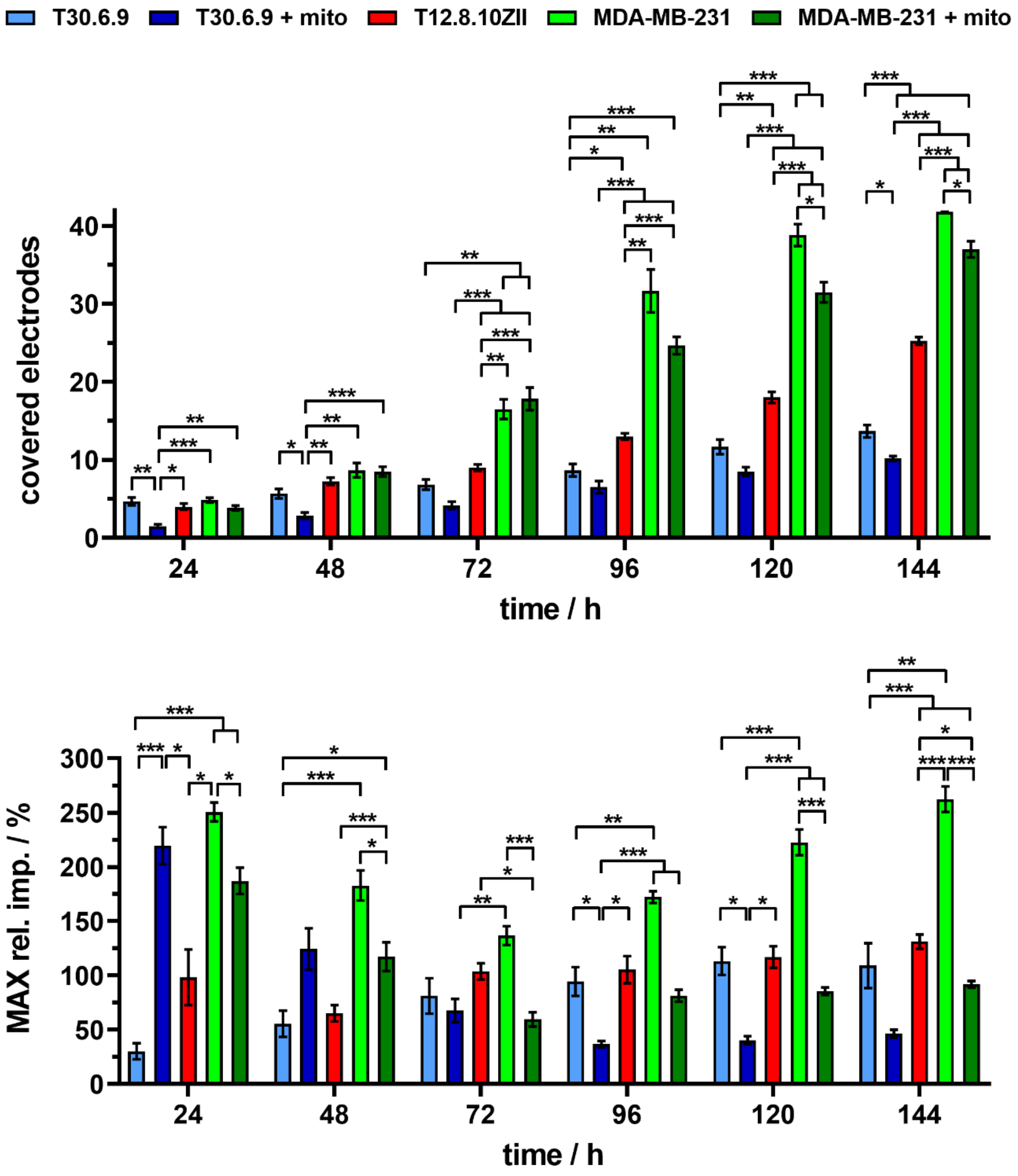

Supplementary Figure S2: Statistical analysis of covered electrodes and maximum relative impedance. (n = 6, for T12.8.10ZII n = 4; mean ± s.e.m.; * P < 0.05; ** P < 0.001; *** P < 0.001)

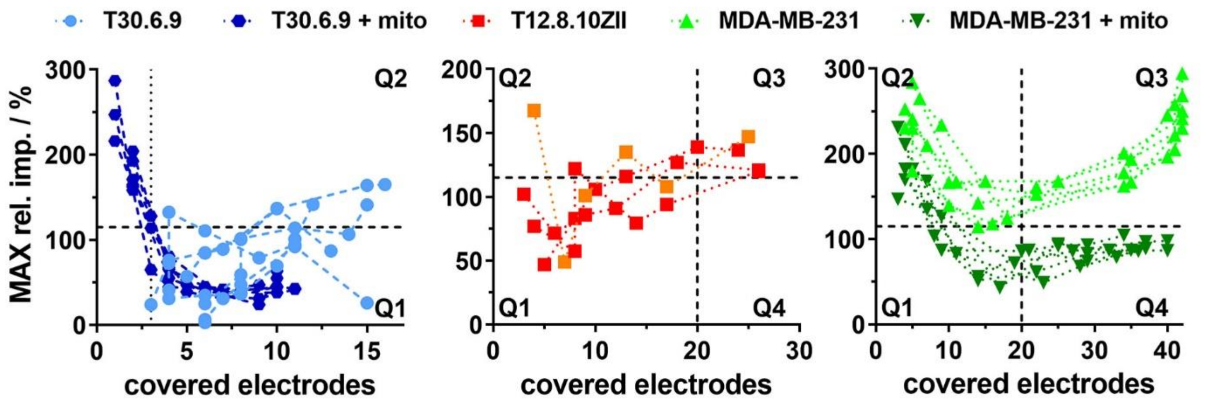

Supplementary Figure S3: Maximum relative impedance values plotted against covered electrodes for individual samples. Maximum relative impedance values represent median for all cell covered electrodes. All groups are represented by six samples, except for T12.8.10ZII, which is represented by four samples. The small doted dashed line at three electrodes (x-axis) in the graph of T30.6.9 samples marks the lowest start value of T30.6.9 samples without mitomycin-C treatment. The orange marked sample in the T12.8.10ZII group (red) is the sample that was linked to the MDA-MB-231 + mito cluster by the cluster analysis (see Fig. 5D).
